# Integrated multidomain screening for geriatric vulnerability in primary care: a conceptual framework for community-dwelling older adults

**DOI:** 10.3389/fpubh.2026.1968310

**Published:** 2026-10-23

**Authors:** Martha P. Arellano-Salazar, Gustavo A. Abad-Fernandez, Karen V. Quiroz Cornejo, Manuel S. Quispe-Villanueva, Víctor J. Samillan

**Affiliations:** 1 Universidad Le Cordon Bleu, Lima, Peru; 2 Facultad de Ciencias, Universidad Nacional del Santa, Chimbote, Peru

**Keywords:** anemia, cardiometabolic health, community-dwelling older adults, geriatric vulnerability, multidomain screening, nutritional risk, primary health care, sarcopenia

## Abstract

**Background:**

Older adults commonly experience overlapping anemia, malnutrition, functional decline, cognitive impairment, and cardiometabolic abnormalities. In primary care, these concerns are often assessed separately, which may limit recognition of multidimensional vulnerability.

**Objective:**

To develop a conceptual methodological framework for integrated multidomain screening of geriatric vulnerability in community-dwelling older adults.

**Methods:**

A 5-step framework was developed by integrating brief screening instruments and point-of-care measurements selected for clinical relevance, feasibility, and previous use in older populations. The framework includes capillary hemoglobin, capillary glucose, blood pressure, waist and calf circumferences, the calf circumference-based Mini Nutritional Assessment–Short Form, SARC-F, and the Rowland Universal Dementia Assessment Scale (RUDAS) or its Peruvian adaptation (PE-RUDAS) when cognitive screening is indicated.

**Results:**

The framework organizes haematologic, nutritional, functional, cognitive, and cardiometabolic screening into 8 conceptual phenotypes using a factorial structure. Nutritional and cardiometabolic findings function as operational modifiers.

**Conclusion:**

The framework is intended to complement, not replace, comprehensive geriatric assessment and other integrated approaches. It does not provide diagnostic or prognostic estimates. Content validation, feasibility, reliability, diagnostic performance, and implementation studies are needed before routine use.

## Introduction

Population aging is accompanied by a growing burden of anemia, malnutrition, functional decline, cognitive impairment, and cardiometabolic abnormalities. These conditions frequently coexist in older adults and may contribute to reduced physiological reserve, disability, multimorbidity, and greater health-care needs. Anemia and malnutrition remain important public health concerns worldwide. In 2021, anemia affected an estimated 24.1% of the global population, corresponding to approximately 1.92 billion cases, and was associated with 52 million disability-adjusted life years; dietary iron deficiency was the leading cause (1). Older adults are particularly vulnerable, with community-based prevalence estimates of 11.0% among men and 10.2% among women aged 65 years or older (2).

In Peru, anemia affects a substantial proportion of older adults and is more frequent among people living in rural areas, in poverty, and at higher altitudes. National estimates have suggested a prevalence of approximately 23.3% among older Peruvian adults, with higher levels reported in regions such as Ayacucho and Ancash (3). In metropolitan Lima, anemia has been reported in 26.7% of non-institutionalized older adults (4). At the same time, population aging is associated with an increasing burden of cardiometabolic disorders and diverse forms of malnutrition. In northern Peru, metabolic syndrome has been reported in 55% of adults and 73% of older adults, highlighting the coexistence of metabolic and nutritional challenges in this population (5, 6).

In routine primary care, haematologic, nutritional, functional, cognitive, and cardiometabolic concerns are often assessed separately. This fragmented approach may delay recognition of co-occurring vulnerabilities and limit opportunities for timely confirmatory assessment or comprehensive evaluation. Brief instruments are available for individual domains, including portable hemoglobin measurement, the Mini Nutritional Assessment–Short Form, the SARC-F questionnaire, the Rowland Universal Dementia Assessment Scale, and basic cardiometabolic measurements. However, their use in isolation may not provide a coherent overview of multidimensional vulnerability in older adults living in the community (7–10).

Existing integrated approaches are essential for appropriately positioning the present proposal. The World Health Organization Integrated Care for Older People (ICOPE) framework provides a person-centered approach for identifying declines in intrinsic capacity and initiating care pathways in primary care and community settings. Comprehensive geriatric assessment offers a broader multidimensional evaluation of medical, functional, cognitive, nutritional, psychological, and social needs. Frailty measures, including the Fried phenotype, frailty indices, the Clinical Frailty Scale, and the Edmonton Frail Scale, identify frailty or cumulative deficits using different conceptual and operational approaches. The Multidimensional Prognostic Index is also based on comprehensive geriatric assessment and has been evaluated against mortality outcomes. Therefore, the present framework does not address an absolute absence of multidomain assessment tools; rather, it proposes a complementary preliminary configuration that incorporates point-of-care haematologic and cardiometabolic screening with brief nutritional, functional, and cognitive assessments (11–15).

The proposed framework is positioned upstream of comprehensive geriatric assessment. Its purpose is not to diagnose anemia, sarcopenia, dementia, diabetes, hypertension, malnutrition, or frailty; nor is it intended to provide prognostic estimates or replace clinical judgment. Instead, it aims to organize brief screening findings into a sequential operational pathway that may help primary care and community teams identify older adults who could benefit from confirmatory testing, closer follow-up, or more comprehensive geriatric evaluation.

## Methods

### Conceptual framework development

This study presents a conceptual methodological framework for multidomain screening of geriatric vulnerability in community-dwelling older adults. The framework was designed to integrate brief screening instruments and point-of-care measurements into a sequential strategy suitable for primary care and community settings. It is not intended to establish clinical diagnoses, predict clinical outcomes, or replace comprehensive geriatric assessment, confirmatory testing, or professional clinical judgment.

### Development of the methodological proposal

The framework was developed through the reasoned integration of previously validated screening tools relevant to haematologic, nutritional, functional, cognitive, and cardiometabolic domains. Instrument selection prioritized clinical relevance, feasibility in community settings, minimal technological requirements, ease of administration, and applicability to older populations.

This work did not involve recruitment of participants, collection of identifiable personal data, biological samples, or interventions in humans. Therefore, institutional ethics review and informed consent were not required. Because no empirical cohort was included, no sample-size calculation, hypothesis testing, regression modeling, effect-size estimation, or diagnostic performance analysis was undertaken. The proposed scores, operational categories, and phenotype ranges should therefore be interpreted as conceptual conventions rather than statistically derived parameters.

### Criteria for the selection of components

Candidate components were selected through a non-systematic narrative appraisal of international guidelines, consensus documents, validation studies, and relevant reviews. The selection was guided by 5 criteria: (1) relevance for identifying common and clinically important conditions in older adults; (2) feasibility in primary care and community settings; (3) availability of brief instruments or point-of-care measurements; (4) previous evidence of use or validation in older populations; and (5) complementarity across haematologic, nutritional, functional, cognitive, and cardiometabolic domains.

Because a formal systematic review and structured Delphi consensus process were not conducted, the selection process may not have identified all relevant instruments and may have introduced selection bias. Formal evidence synthesis and expert consensus are therefore recommended as subsequent phases of framework refinement.

### Intended scope of the algorithm

The framework was designed to support initial screening and preliminary clinical orientation in community-dwelling older adults. It is not intended to diagnose anemia, diabetes, hypertension, cognitive impairment, sarcopenia, malnutrition, or frailty. Rather, it aims to organize available screening findings and identify individuals who may require confirmatory testing, closer follow-up, or comprehensive geriatric assessment.

The framework has 2 intended applications: first, to provide a structured sequence for initial multidomain screening in primary care and community settings; and second, to offer a reproducible methodological basis for future validation and implementation research. Instruments were prioritized when they could be administered by trained primary care or community health personnel within a brief encounter, interpreted without advanced diagnostic infrastructure, and used as screening rather than definitive diagnostic tools.

### Framework integration

The framework was organized as a sequential pathway that prioritizes low-cost, feasible assessments. The sequence begins with point-of-care measurements and basic anthropometry, followed by nutritional and functional screening, and cognitive screening when clinically indicated. The available findings are then integrated into either a complete multidomain screening profile or a provisional profile when a required domain has not been assessed. Missing information is documented explicitly and is not interpreted as a normal result.

#### Portable hemoglobin measurement

Portable hemoglobinometers and other point-of-care devices are commonly used to measure hemoglobin concentration in field and resource-limited settings. The HemoCue system has been widely used for capillary hemoglobin assessment in rural and low-resource environments, whereas the EKF Hemo Control analyzer has shown agreement with automated analyzers in complex settings, including high-altitude areas (8, 16–19). Capillary hemoglobin values may be affected by hydration status, peripheral perfusion, systemic inflammation, and sampling technique. Interpretation should therefore account for sex, altitude, the guideline adopted by the implementation setting, and pre-analytical conditions of capillary sampling (20, 21). The framework does not define a universal hemoglobin threshold. Instead, screening thresholds should be prespecified according to sex and adjusted for altitude when applicable. Any abnormal screening result should prompt confirmatory clinical evaluation and, where feasible, laboratory testing using locally applicable diagnostic procedures.

#### Nutritional evaluation

Nutritional status was assessed using the Mini Nutritional Assessment and its Short Form (MNA-SF), which are widely used and validated instruments for older adults. The calf circumference-based MNA-SF (CC-MNA-SF) is particularly useful when body weight and height cannot be reliably obtained, such as in community or resource-limited settings, while maintaining diagnostic performance comparable to the body mass index-based version (22–24).

In the present framework, calf circumference was interpreted using the calf circumference cut-off of 31 cm. The CC-MNA-SF classifies individuals as having normal nutritional status, being at risk of malnutrition, or having malnutrition. To facilitate transparent organization of the conceptual matrix, operational weights of 0, 1, and 3 were assigned to these categories, respectively. These weights were introduced only to illustrate combinations of screening findings and do not represent calibrated measures of disease severity, prognosis, urgency, or treatment priority. They have not been derived from outcome modeling, formal expert consensus, or validation against reference standards. Their appropriateness should be assessed through content validation, including structured expert consensus, before empirical evaluation of the framework.

#### Functional–musculoskeletal evaluation

The SARC-F questionnaire was included as a brief instrument for identifying positive sarcopenia screening results. It assesses strength, assistance with walking, rising from a chair, climbing stairs, and falls during the previous year (25). Within this framework, a SARC-F score of 4 or higher indicates a positive screening result and should prompt further assessment of muscle strength and, when feasible, muscle quantity or quality and physical performance according to applicable consensus recommendations. A positive SARC-F result alone should not be interpreted as a diagnosis of probable or confirmed sarcopenia (26, 27).

#### Cognitive component

Cognitive screening was considered when functional deterioration, advanced age, low educational attainment, illiteracy, or clinical concern for neurological impairment was present. The Rowland Universal Dementia Assessment Scale (RUDAS) and its Peruvian adaptation (PE-RUDAS) were selected because of their suitability for individuals with low educational attainment or limited literacy. A score below 23 was considered suggestive of possible cognitive impairment and should be followed by confirmatory clinical assessment (28–30).

Because cognitive screening is not applied universally within the framework, individuals without a cognitive assessment should be classified as having a provisional multidomain screening profile with cognitive assessment pending. They should not be assigned to 1 of the 8 complete phenotypes. The reason for unavailable cognitive screening should be recorded.

#### Cardiometabolic screening

The cardiometabolic component includes capillary blood glucose, waist circumference, and blood pressure. Capillary glucose may be useful for initial screening where laboratory testing is not readily available, but it does not replace confirmatory diagnostic testing (31–33). Before interpreting capillary glucose, personnel should record whether the participant has fasted for at least 8 h and document the time since the last caloric intake. Fasting and non-fasting measurements should be interpreted using separate prespecified screening pathways, and abnormal findings should be confirmed through locally applicable laboratory or clinical procedures.

Waist circumference was included as an indicator of abdominal adiposity, using cut-offs of at least 94 cm in men and at least 88 cm in women (34–37). Blood pressure was considered elevated at values of at least 140/90 mmHg or in participants with a previous diagnosis of hypertension receiving antihypertensive treatment (38, 39).

One operational point was assigned to each altered cardiometabolic screening parameter, resulting in a total score from 0 to 3. This score is not a validated cardiovascular or metabolic prediction model; it is a preliminary convention intended to organize screening findings and support prioritization of confirmatory assessment. The terms low, moderate, and high refer only to operational categories within this conceptual framework and do not estimate the absolute or relative risk of future clinical events.

#### Operational sequence of the proposed model

The framework includes 5 sequential steps: (1) point-of-care assessment of capillary hemoglobin, capillary blood glucose, and blood pressure; (2) nutritional and anthropometric screening, including waist circumference, calf circumference, and CC-MNA-SF; (3) functional screening with SARC-F; (4) cognitive screening with RUDAS or PE-RUDAS when clinically indicated; and (5) integration of available findings into a complete or provisional multidomain screening profile. The sequential workflow is presented in Figure 1.

**Figure 1:**
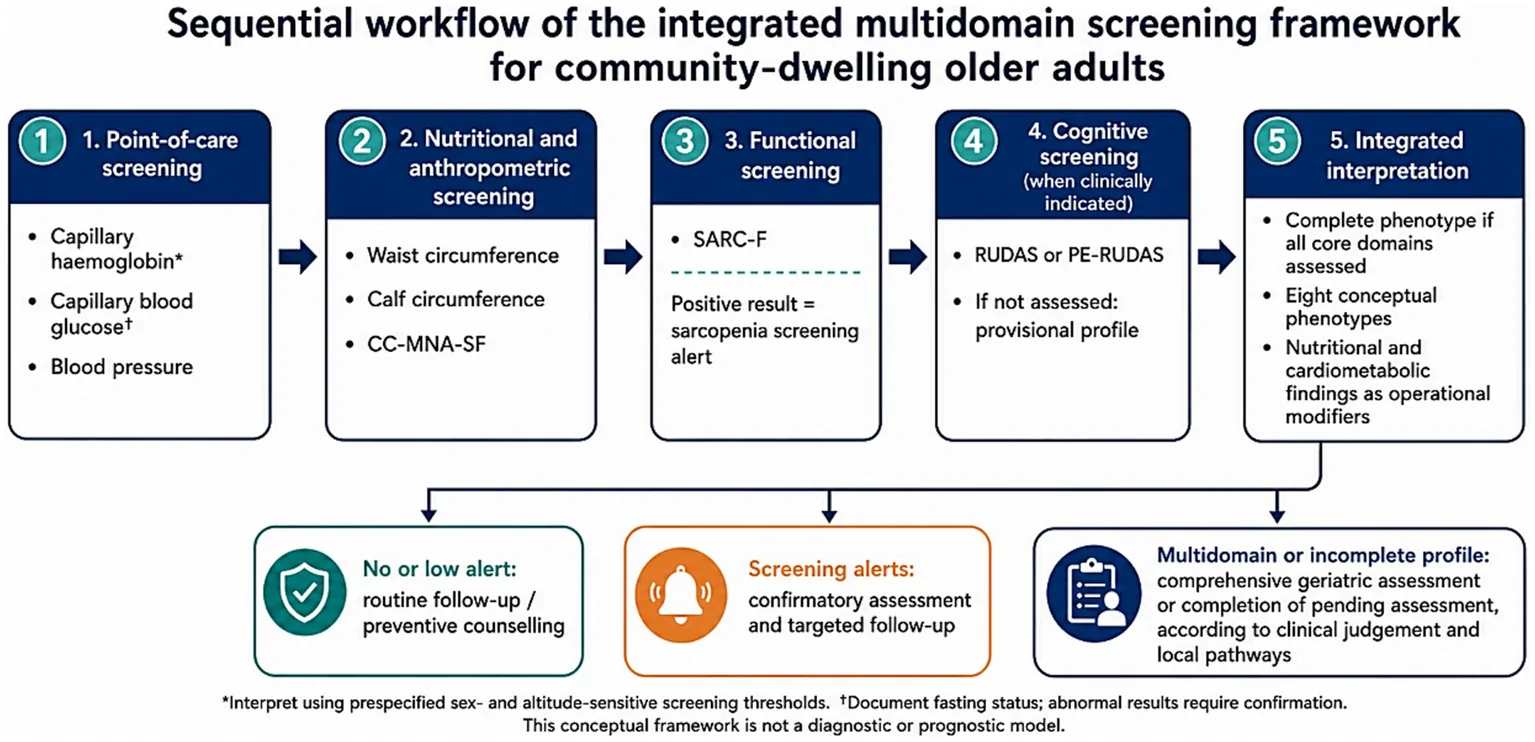
Sequential workflow of the integrated multidomain screening framework for community-dwelling older adults. The framework begins with point-of-care haematologic and cardiometabolic screening, followed by nutritional, anthropometric, functional, and, when clinically indicated, cognitive assessment. Available findings are integrated into either a complete multidomain profile or a provisional profile when cognitive assessment is pending. The framework is intended to organize initial screening findings and guide confirmatory assessment or follow-up according to clinical judgment and local care pathways. It does not constitute a validated diagnostic, prognostic, or therapeutic decision model.

Table 1 summarizes the operational definitions, screening categories, and illustrative weights used in the framework. The weights are conceptual conventions and should not be interpreted as validated measures of diagnostic probability, prognosis, referral priority, or treatment need.

**Table 1:** Operational definitions and illustrative scoring conventions for the multidomain screening framework.

Domain	Screening result	Operational category	Illustrative score
Capillary hemoglobin screening	At or above the prespecified sex- and altitude-sensitive screening threshold	No haematologic screening alert	0
Capillary hemoglobin screening	Below the prespecified sex- and altitude-sensitive screening threshold	Haematologic screening alert	2
SARC-F	0–3 points	No sarcopenia screening alert	0
SARC-F	≥4 points	Positive sarcopenia screening result	2
RUDAS or PE-RUDAS*	23–30 points	No suspected cognitive impairment	0
RUDAS or PE-RUDAS*	≤22 points	Suspected cognitive impairment	2
CC-MNA-SF	12–14 points	Normal nutritional status	0
CC-MNA-SF	8–11 points	At risk of malnutrition	1
CC-MNA-SF	0–7 points	Malnutrition	3
Cardiometabolic screening	0 altered parameters	No altered cardiometabolic screening parameter	0
Cardiometabolic screening	1 altered parameter	1 altered cardiometabolic screening parameter	1
Cardiometabolic screening	2 altered parameters	2 altered cardiometabolic screening parameters	2
Cardiometabolic screening	3 altered parameters	3 altered cardiometabolic screening parameters	3

*PE-RUDAS, Peruvian adaptation of RUDAS; CC-MNA-SF, calf circumference-based Mini Nutritional Assessment–Short Form; RUDAS, Rowland Universal Dementia Assessment Scale; SARC-F, strength, assistance with walking, rise from a chair, climb stairs, and falls.

#### Construction of the factorial matrix

To make the internal organization of the framework transparent, a factorial matrix was generated from the predefined categories of the 5 screening domains. The matrix includes 96 theoretical combinations, derived from 2 hematologic screening categories, 2 sarcopenia screening categories, 2 cognitive categories, 4 cardiometabolic screening categories, and 3 nutritional categories. The factorial enumeration was used as an organizational device rather than as a derived clinical classification, empirical finding, or prediction model.

For interpretability, the combinations were grouped into 8 conceptual phenotypes defined by haematologic screening status, sarcopenia screening status, and cognitive screening status. Nutritional status and cardiometabolic screening were retained as operational modifiers within each phenotype. The matrix was generated using IBM SPSS Statistics version 32 and computationally verified to confirm that no theoretical combinations were duplicated or omitted.

### Methodological limitations of the proposal

This framework has several methodological limitations. The selected domains, screening thresholds, operational weights, and phenotype labels were not derived from clinical outcome data, formal systematic review methods, or structured expert consensus. The framework has not undergone content validation, feasibility testing, acceptability assessment, inter-rater reliability analysis, diagnostic-accuracy evaluation, calibration assessment, external validation, or implementation evaluation.

Accordingly, the proposed phenotypes, scores, and operational categories should be interpreted as preliminary conceptual conventions. They do not constitute diagnostic criteria, prognostic estimates, referral mandates, or therapeutic recommendations. Future work should include structured expert consensus, pilot feasibility studies in primary care, reliability assessment, comparison with appropriate reference standards, and prospective evaluation of diagnostic performance and implementation outcomes.

## Results

### Algorithm structure and theoretical classification matrix

The main output of this conceptual work was an integrated screening framework that combines haematologic, nutritional, functional, cognitive, and cardiometabolic screening within a single sequential pathway. The framework does not derive from observations in a clinical cohort; rather, it organizes predefined categories from established screening instruments and point-of-care measurements.

The factorial structure generated 96 theoretical combinations across the 5 screening domains. These combinations were derived from 2 haematologic screening categories, 2 sarcopenia screening categories, 2 cognitive categories, 4 cardiometabolic screening categories, and 3 nutritional categories. The resulting configurations were grouped into 8 conceptual phenotypes according to the 3 principal domains: haematologic screening status, sarcopenia screening status, and cognitive screening status. Nutritional status and cardiometabolic screening were retained as operational modifiers within each phenotype.

The 8 phenotypes are summarized in Table 2. The complete factorial matrix of 96 theoretical combinations, including the 12 nutritional–cardiometabolic subcategories within each phenotype, is provided in Supplementary Table S1. The phenotypes and their associated operational scores are intended to describe the internal organization of the framework. They should not be interpreted as clinical diagnoses, validated risk categories, prognostic estimates, or treatment-allocation criteria (40–42).

**Table 2:** Conceptual phenotypes based on haematologic, sarcopenia screening, and cognitive screening findings.

Hemoglobin	Sarcopenia	Cognitive screening status	Phenotype name	Score range
Normal	Low alert	Normal	Preserved profile	0–6
Normal	Low alert	Suspected cognitive impairment	Cognitive alert (C-Flag)	2–8
Normal	High alert	Normal	Muscle alert (S-Flag)	2–8
Low	Low alert	Normal	Haematologic alert (H-Flag)	2–8
Normal	High alert	Suspected cognitive impairment	Muscle–cognitive alert (SC-Flag)	4–10
Low	Low alert	Suspected cognitive impairment	Haematologic–cognitive alert (HC-Flag)	4–10
Low	High alert	Normal	Haematologic–muscle alert (HS-Flag)	4–10
Low	High alert	Suspected cognitive impairment	Multidomain alert (HSC-Flag)	6–12

*Score ranges are illustrative organizational conventions and do not represent validated measures of disease severity, prognosis, or referral priority.

### Alert classification according to vulnerability phenotypes

The preserved profile includes no haematologic screening alert, no positive sarcopenia screening result, and no suspected cognitive impairment. Nutritional status and cardiometabolic screening are recorded as operational modifiers. This profile does not establish the absence of disease or future vulnerability; it reflects only the screening findings included in the proposed framework. General health maintenance, nutritional counseling, and regular monitoring are consistent with healthy aging approaches in older adults (26, 43) (Supplementary Table S2).

The cognitive alert phenotype includes suspected cognitive impairment in the absence of a haematologic screening alert or a positive sarcopenia screening result. Nutritional and cardiometabolic screening may be recorded as operational modifiers. Cognitive impairment in older adults may coexist with nutritional concerns, vascular factors, social isolation, and reduced food intake; however, the framework does not determine the cause, severity, or prognosis of cognitive impairment (44–46) (Supplementary Table S3).

The muscle alert phenotype includes a positive sarcopenia screening result according to the SARC-F questionnaire in the absence of a haematologic screening alert or suspected cognitive impairment. A positive SARC-F result indicates the need for further functional assessment and does not establish a diagnosis of sarcopenia (25, 26). Nutritional risk, low physical activity, adiposity, and cardiometabolic abnormalities may coexist with muscle impairment in older adults (36, 47, 48) (Supplementary Table S4).

The haematologic alert phenotype includes a haematologic screening alert in the absence of a positive sarcopenia screening result or suspected cognitive impairment. A low capillary hemoglobin screening result may be associated with nutritional deficiencies, inflammation, chronic kidney disease, occult blood loss, or other clinical conditions; the framework does not identify the etiology of the hematologic finding (49–51) (Supplementary Table S5).

The muscle–cognitive alert phenotype includes a positive sarcopenia screening result and suspected cognitive impairment. The coexistence of functional and cognitive concerns may occur alongside nutritional vulnerability, reduced physical activity, depressive symptoms, and cardiometabolic abnormalities, although the present framework does not establish causal relationships between these findings (42, 52–54) (Supplementary Table S6).

The haematologic–cognitive alert phenotype includes a haematologic screening alert and suspected cognitive impairment, without a positive sarcopenia screening result. Anemia may be associated with cognitive impairment through multiple pathways, including reduced oxygen delivery and reduced cerebrovascular reserve in susceptible older adults. Nutritional deficiencies, chronic kidney disease, and vascular conditions may also contribute to the coexistence of these findings (4, 55, 56). The framework does not establish whether these findings share a common cause or predict subsequent cognitive, functional, or clinical outcomes (Supplementary Table S7).

The haematologic–muscle alert phenotype includes a haematologic screening alert and a positive sarcopenia screening result, without suspected cognitive impairment. Anemia and muscle impairment may coexist in older adults with nutritional deficits, chronic disease, inflammation, low physical activity, or other underlying conditions (57, 58). The framework does not establish causal mechanisms, disease severity, or treatment priorities (Supplementary Table S8).

The multidomain alert phenotype includes a haematologic screening alert, a positive sarcopenia screening result, and suspected cognitive impairment. This profile represents the most complex configuration within the conceptual framework because screening findings are present across all 3 principal domains. Nutritional vulnerability and cardiometabolic abnormalities may further characterize this profile. The coexistence of these conditions has been associated in previous literature with lower physiological reserve and greater multidimensional needs; however, the framework does not diagnose frailty, predict adverse outcomes, or prescribe a specific management pathway (59–63) (Supplementary Table S9).

### Interpretation of operational categories

Across the 8 phenotypes, nutritional status and cardiometabolic screening were used to organize the 12 subcategories within each phenotype. The numerical values assigned to individual screening categories were included solely to provide a transparent and consistent structure for the factorial matrix. They do not represent calibrated measures of clinical severity, probability of disease, prognosis, urgency of referral, or expected response to treatment (40–42). The conceptual framework distinguishes between complete and provisional screening profiles. A complete profile requires assessment of the 3 principal domains: haematologic status, sarcopenia screening status, and cognitive screening status. When cognitive assessment is not performed, the framework records a provisional profile rather than assigning a complete phenotype. This approach preserves transparency regarding missing information and avoids treating an unassessed domain as normal.

### Application in community and primary care setting

The framework is intended to support initial organization of multidomain screening findings in primary care and community settings. It may facilitate systematic documentation of available information and identify situations in which confirmatory assessment, closer follow-up, or comprehensive geriatric evaluation should be considered according to clinical judgment and local care pathways. The framework does not replace diagnostic assessment, comprehensive geriatric assessment, or established clinical guidelines (26, 59, 62). Figure 1 presents the sequential workflow of the framework, from point-of-care and anthropometric assessment to functional screening, cognitive screening when clinically indicated, and integration into a complete or provisional multidomain profile. Table 1 provides the operational definitions and illustrative weights for each screening domain, while Table 2 summarizes the 8 conceptual phenotypes.

## Discussion

This conceptual framework proposes a sequential approach for organizing haematologic, nutritional, functional, cognitive, and cardiometabolic screening in community-dwelling older adults. Its purpose is not to create a new diagnostic instrument or to provide a validated prognostic model. Rather, it provides a preliminary structure that may help primary care and community teams document multidomain findings and identify individuals who may require confirmatory testing, closer follow-up, or comprehensive geriatric assessment.

The proposed framework should be interpreted in relation to established integrated approaches rather than as a replacement for them. The World Health Organization Integrated Care for Older People framework provides person-centered guidance for identifying declines in intrinsic capacity and initiating care pathways in primary care and community settings. Comprehensive geriatric assessment offers a broader multidimensional evaluation that includes medical, functional, cognitive, nutritional, psychological, and social domains. Frailty measures, including the Fried phenotype, frailty indices, the Clinical Frailty Scale, and the Edmonton Frail Scale, use different conceptual and operational approaches to identify frailty or accumulated deficits. The Multidimensional Prognostic Index differs from the present proposal because it is derived from comprehensive geriatric assessment and has been evaluated against mortality outcomes. The present framework is therefore positioned upstream of comprehensive geriatric assessment and as complementary to ICOPE and frailty assessment, rather than as an alternative diagnostic or prognostic tool (11–15, 59).

The specific contribution of the framework lies in combining point-of-care haematologic and cardiometabolic screening with brief nutritional, functional, and cognitive assessments in a single initial workflow. This configuration may be relevant in primary care or community programs where consultation time, workforce capacity, and access to specialized diagnostic services are limited. The explicit distinction between complete and provisional profiles is also intended to prevent unassessed domains, particularly cognition, from being interpreted as normal. The potential feasibility, acceptability, added value, and effect on clinical pathways of this approach require empirical evaluation.

The factorial matrix was designed to make the internal organization of the framework transparent. The 96 theoretical combinations are a descriptive enumeration of predefined categories, not an empirically derived classification or a principal outcome of the study. The central contribution of the proposal is the structured sequencing and integration of the selected domains. The 8 phenotypes provide an interpretable way to organize combinations of haematologic screening status, sarcopenia screening status, and cognitive screening status, while nutritional and cardiometabolic findings function as operational modifiers. The assigned numerical values do not represent calibrated measures of disease severity, clinical risk, prognosis, referral urgency, or expected response to treatment (40–42). The selected domains are biologically and clinically relevant in older populations. Anemia, nutritional impairment, muscle dysfunction, cognitive impairment, and cardiometabolic abnormalities may coexist through shared pathways involving chronic disease, inflammation, reduced physical activity, nutritional deficits, vascular factors, and social vulnerability. Anemia may be associated with reduced exercise tolerance and cognitive impairment, whereas nutritional risk and cardiometabolic abnormalities may coexist with functional decline and muscle impairment (36, 49, 51, 56–58). However, the present framework does not establish causal relationships between these domains. Its purpose is to organize co-occurring screening findings and not to identify their etiology, temporal sequence, or clinical consequences.

The framework may have relevance for public health planning because multidomain vulnerability is often identified through separate assessments conducted at different times or in different services. A structured initial workflow could support consistent documentation of available screening findings and facilitate consideration of integrated follow-up pathways in community and primary care settings. This potential role is particularly relevant in settings with geographic, socioeconomic, and resource constraints, including rural and high-altitude areas where access to specialized care may be limited (3, 8). Nevertheless, the framework has not been evaluated for feasibility, cost, equity, health-system impact, or patient outcomes.

Several limitations should be considered. The component selection, screening thresholds, numerical weights, operational categories, and phenotype labels were based on a non-systematic narrative appraisal and reasoned clinical judgment rather than systematic evidence synthesis, formal expert consensus, empirical outcome modeling, or diagnostic validation. The framework has not undergone content validation, feasibility testing, inter-rater reliability assessment, diagnostic-accuracy evaluation, calibration analysis, external validation, or implementation research. In addition, capillary hemoglobin and glucose measurements, anthropometric indicators, and brief screening tools may be influenced by training, measurement conditions, sex, altitude, comorbidity, and access to confirmatory testing. These limitations preclude the use of the framework as a diagnostic, prognostic, referral, or treatment decision tool.

Future development should proceed in stages. First, a structured expert-consensus process, such as a Delphi study, should assess the relevance, clarity, completeness, feasibility, and acceptability of the selected domains, thresholds, weights, and operational categories. Second, pilot studies should evaluate completion time, training requirements, acceptability, inter-rater reliability, and integration within primary care and community workflows. Third, prospective studies should compare the framework with appropriate reference standards and assess diagnostic performance, calibration, discrimination, and clinical utility. Finally, implementation studies should examine effects on service use, referral patterns, equity, patient-centered outcomes, and costs before the framework is considered for routine application.

## Conclusion

This conceptual framework integrates brief haematologic, nutritional, functional, cognitive, and cardiometabolic screening components into a sequential approach for community-dwelling older adults. It is intended to reduce fragmentation in initial screening and to provide a transparent structure for organizing multidomain findings in primary care and community settings.

The framework is positioned as complementary to established approaches, including comprehensive geriatric assessment, frailty assessment, and integrated care pathways. It does not establish diagnoses, predict clinical outcomes, or replace confirmatory assessment, clinical judgment, or locally applicable care pathways. The proposed phenotypes, numerical weights, and operational categories are conceptual conventions and should not be interpreted as validated measures of risk, severity, prognosis, referral priority, or treatment need.

Further work should assess content validity, feasibility, reliability, diagnostic performance, and implementation outcomes before this framework can be considered for routine clinical or public health use. Future studies should also examine its potential value for supporting integrated care for older adults in resource-limited, rural, and high-altitude settings (3, 8, 21).

### Contribution to the field

This manuscript presents a conceptual framework for integrating brief haematologic, nutritional, functional, cognitive, and cardiometabolic screening components in community-dwelling older adults. Rather than introducing a new diagnostic or prognostic instrument, the framework provides a structured, sequential approach for organizing multidomain screening findings in primary care and community settings.

The proposal is intended to complement established approaches, such as comprehensive geriatric assessment, frailty assessment, and integrated care pathways, by incorporating point-of-care hemoglobin and cardiometabolic measurements alongside brief geriatric screening tools. Its specific contribution is the integration of these domains into a transparent framework that distinguishes between complete and provisional profiles when cognitive assessment is unavailable.

The framework has not been empirically validated and should not be used as a diagnostic, prognostic, or treatment decision tool. Nevertheless, it offers a methodological basis for future content validation, feasibility, diagnostic performance, and implementation studies aimed at improving integrated care for older adults in primary care and community settings.

## Data Availability

The original contributions presented in the study are included in the article/Supplementary material, further inquiries can be directed to the corresponding author.
