# Supplementary material for: Integrated multidomain screening for geriatric vulnerability in primary care: a conceptual framework for community-dwelling older adults
Source: Front Public Health. 2026 Oct 23;14:1968310. doi: 10.3389/fpubh.2026.1968310 (PMC13739804; doi:10.3389/fpubh.2026.1968310)
Supplement: Supplementary file 1 [file Supplementary_file_1.pdf]

Supplementary material

Table S1. Screening Matrix by Phenotype and Subcategories.

Anaemia	Sarcopenia	Cognitive Impairment	Phenotype Category	No Cardiometabolic screening			Low Cardiometabolic screening			Moderate Cardiometabolic screening			High Cardiometabolic screening		
				Normal	Alert of Malnutrition	Established Malnutrition	Normal	Alert of Malnutrition	Established Malnutrition	Normal	Alert of Malnutrition	Established Malnutrition	Normal	Alert of Malnutrition	Established Malnutrition
No	No	No	Preserved	0	1	3	1	2	4	2	3	5	3	4	6
No	No	Yes	C-Flag	2	3	5	3	4	6	4	5	7	5	6	8
No	Yes	No	S-Flag	2	3	5	3	4	6	4	5	7	5	6	8
Yes	No	No	H-Flag	2	3	5	3	4	6	4	5	7	5	6	8
No	Yes	Yes	SC-Flag	4	5	7	5	6	8	6	7	9	7	8	10
Yes	No	Yes	HC-Flag	4	5	7	5	6	8	6	7	9	7	8	10
Yes	Yes	No	HS-Flag	4	5	7	5	6	8	6	7	9	7	8	10
Yes	Yes	Yes	HSC-Flag	6	7	9	7	8	10	8	9	11	9	10	12

Alert Category Legend

No alert -	Low alert +	Moderate alert ++	High alert +++	Very high alert ++++

Supplementary Table S2. Phenotype 1 or Preserved Profile

<table>
<tr><td colspan="2">Capillary haemoglobin</td><td colspan="3">Normal</td><td>0</td></tr>
<tr><td colspan="2">Sarcopenia (SARC-F)</td><td colspan="3">Low alert</td><td>0</td></tr>
<tr><td colspan="2">Cognitive screening status (RUDAS)</td><td colspan="3">Normal</td><td>0</td></tr>
<tr><td>Sub *</td><td>Alert CM</td><td>Nutritional Status CC-MNA-SF</td><td>Score</td><td>Alert class.</td><td>Conceptual Interpretation</td></tr>
<tr><td>1</td><td>No alert</td><td>Normal</td><td>0</td><td>-</td><td>Optimal profile across the five domains</td></tr>
<tr><td>2</td><td>Low alert</td><td>Normal</td><td>1</td><td>+</td><td>Incipient Cardiometabolic screening, with preserved functional/nutritional reserve</td></tr>
<tr><td>3</td><td>No alert</td><td>Alert of malnutrition</td><td>1</td><td>+</td><td>Early nutritional alert</td></tr>
<tr><td>4</td><td>Low alert</td><td>Alert of malnutrition</td><td>2</td><td>+</td><td>Mild dual alert, both conditions potentially modifiable</td></tr>
<tr><td>5</td><td>Moderate alert</td><td>Normal</td><td>2</td><td>+</td><td>Relevant Cardiometabolic screening with preserved functional/nutritional reserve</td></tr>
<tr><td>6</td><td>High alert</td><td>Normal</td><td>3</td><td>+</td><td>Isolated high Cardiometabolic screening with preserved functional/nutritional reserve</td></tr>
</table>

7	Moderate alert	Alert of malnutrition	3	+	Moderate dual alert; caution is required to prevent metabolic management from contributing to malnutrition
8	No alert	Established malnutrition	3	+	Discordant pattern of malnutrition without current metabolic impairment but with future Cardiometabolic screening (Özgün et al. 2026)
9	High alert	Alert of malnutrition	4	++	Condition requiring close monitoring
10	Low alert	Established malnutrition	4	++	Established malnutrition is the priority
11	Moderate alert	Established malnutrition	5	++	Clearly moderate alert, requiring balance between both domains.
12	High Alert	Established malnutrition	6	++	Complex scenario requiring individualized and carefully balanced cardiometabolic and nutritional management.

*: subcategory, Alert classification: no alert: –; low alert: +; moderate alert: ++; high alert: +++; very high alert: ++++. DM2: type 2 diabetes mellitus; HTA: arterial hypertension.

Supplementary Table S3. Phenotype 2 or Cognitive Alert or C-Flag

**Capillary haemoglobin**		Normal			0
**Sarcopenia (SARC-F)**		Low alert			0
**Cognitive screening status (RUDAS)**		Suspected cognitive Impairment			2
**Sub***	**Alert CM**	**Nutritional Status CC-MNA-SF**	**Score**	**Alert Class.**	**Conceptual Interpretation**
1	No alert	Normal	2	+	Isolated cognitive impairment, without additional vascular or nutritional factors
2	Low alert	Normal	3	+	Early possible vascular contribution to cognitive impairment
3	No alert	Alert of malnutrition	3	+	Early nutritional alert that could affect cognition due to micronutrient deficiencies
4	Low alert	Alert of malnutrition	4	++	Mild dual alert, involving vascular and nutritional factors
5	Moderate alert	Normal	4	++	Vascular component already clinically relevant to cognitive decline
6	High alert	Normal	5	++	High Cardiometabolic screening as a significant vascular contributor
7	Moderate alert	Alert of malnutrition	5	++	Mixed vascular–nutritional profile
8	No alert	Established malnutrition	5	++	Established malnutrition as a probable determinant of cognitive

					impairment without a vascular component
9	High alert	Alert of malnutrition	6	++	Convergence of two modifiable factors—vascular and nutritional—affecting cognition
10	Low alert	Established malnutrition	6	++	Established malnutrition is likely the primary driver, with an additional mild vascular alert component
11	Moderate alert	Established malnutrition	7	+++	High-alert profile with two active components: moderate vascular alert and established nutritional impairment
12	High alert	Established malnutrition	8	+++	Complex scenario: cognitive impairment with combined vascular and nutritional components

*: Subcategory. Alert classification: no alert: –; low alert: +; moderate alert: ++; high alert: +++; very high alert: ++++.

Supplementary Table S4. Phenotype 3 or Muscle Alert Phenotype (S-Flag)

**Capillary haemoglobin**		Normal			0
**Sarcopenia (SARC-F)**		High alert			2
**Cognitive screening status (RUDAS)**		Normal			0
**Sub***	**Alert CM**	**Nutritional Status CC-MNA-SF**	**Score**	**Alert Class.**	**Conceptual Interpretation**
1	No alert	Normal	2	+	Isolated sarcopenia without cardiometabolic or nutritional comorbidity
2	Low alert	Normal	3	+	Sarcopenia with early Cardiometabolic screening
3	No alert	Alert of malnutrition	3	+	Sarcopenia with early nutritional alert, likely related to insufficient protein intake
4	Low alert	Alert of malnutrition	4	++	Mild triple alert involving muscular, vascular, and nutritional domains
5	Moderate alert	Normal	4	++	Sarcopenia with moderate Cardiometabolic screening and preserved nutritional status
6	High alert	Normal	5	++	Sarcopenia with high Cardiometabolic screening, a combination associated with increased cardiometabolic morbidity
7	Moderate alert	Alert of malnutrition	5	++	Moderate triple-domain involvement, a condition

					requiring timely intervention
8	No alert	Established malnutrition	5	++	Sarcopenia with Established malnutrition and no Cardiometabolic screening, likely compatible with malnutrition-related secondary sarcopenia
9	High alert	Alert of malnutrition	6	++	Potential therapeutic dilemma due to the need for Cardiometabolic screening reduction while maintaining adequate protein intake
10	Low alert	Established malnutrition	6	++	Established malnutrition is likely the primary cause of muscle loss
11	Moderate alert	Established malnutrition	7	+++	High-alert profile characterized by sarcopenia, Established malnutrition, and moderate Cardiometabolic screening
12	High alert	Established malnutrition	8	+++	Most complex scenario, with the highest alert of cardiometabolic morbidity and mortality compounded by Established malnutrition.

*: Subcategory. Alert classification: no alert: –; low alert: +; moderate alert: ++; high alert: +++; very high alert: ++++.

Supplementary Table S5. Phenotype 4 or Haematologic Alert Phenotype (H-Flag)

**Capillary haemoglobin**		Low			2
**Sarcopenia (SARC-F)**		Low alert			0
**Cognitive screening status (RUDAS)**		Normal			0
**Sub***	**Alert CM**	**Nutritional Status CC-MNA-SF**	**Score**	**Alert Class.**	**Conceptual Interpretation**
1	No alert	Normal	2	+	Isolated anaemia without cardiometabolic or nutritional comorbidity
2	Low alert	Normal	3	+	Anaemia with early Cardiometabolic screening
3	No alert	Alert of malnutrition	3	+	Anaemia with early nutritional alert, suggesting a possible shared nutritional aetiology (iron, folate, or vitamin B12 deficiency)
4	Low alert	Alert of malnutrition	4	++	Mild dual alert, with both conditions potentially sharing a nutritional origin
5	Moderate alert	Normal	4	++	Moderate Cardiometabolic screening as a possible active contributor to anaemia (anaemia of chronic disease)

6	High alert	Normal	5	++	High Cardiometabolic screening and concurrent anaemia, resulting in a poorer cardiovascular prognosis
7	Moderate alert	Alert of malnutrition	5	++	Mixed profile. In older adults, anaemia frequently has a combined inflammatory and nutritional aetiology
8	No alert	Established malnutrition	5	++	Anaemia with Established malnutrition and no Cardiometabolic screening, a pattern consistent with a purely nutritional cause
9	High alert	Alert of malnutrition	6	++	Anaemia with both cardiometabolic and nutritional contributing factors
10	Low alert	Established malnutrition	6	++	Established malnutrition is likely the primary cause of anaemia
11	Moderate alert	Established malnutrition	7	+++	High-alert profile characterized by anaemia, Established malnutrition, and moderate Cardiometabolic screening
12	High alert	Established malnutrition	8	+++	The most complex scenario within this phenotype

*: Subcategory. Alert classification: no alert: –; low alert: +; moderate alert: ++; high alert: +++; very high alert: ++++.

Supplementary Table S6. Phenotype 5 or Muscle–Cognitive Alert Phenotype (SC-Flag)

**Capillary haemoglobin**		Normal			0
**Sarcopenia (SARC-F)**		High alert			2
**Cognitive screening status (RUDAS)**		Suspected cognitive impairment			2
**Sub** *	**Alert CM**	**Nutritional status CC-MNA-SF**	**Score**	**Alert class.**	**Conceptual Interpretation**
1	No alert	Normal	4	++	Sarcopenia and cognitive impairment without cardiometabolic or nutritional comorbidity, a profile consistent with chronic low-grade inflammation (Dyer et al. 2024; Antuña et al. 2022).
2	Low alert	Normal	5	++	An early vascular component is superimposed on the underlying muscular and cognitive impairment.
3	No alert	Alert of malnutrition	5	++	Nutritional alert emerges as a third contributing factor, further compromising both muscle function and cognitive performance.
4	Low alert	Alert of malnutrition	6	++	Early, generalized impairment suggestive

					of an evolving systemic process.
5	Moderate alert	Normal	6	++	Moderate Cardiometabolic screening may accelerate progression in both already compromised domains.
6	High alert	Normal	7	+++	High Cardiometabolic screening is superimposed on the existing muscle and cognitive impairment, potentially accelerating both processes and promoting progression toward greater frailty.
7	Moderate alert	Alert of malnutrition	7	+++	The coexistence of four compromised domains, even at moderate severity, May represent a configuration warranting further evaluation; longitudinal implications require empirical study.
8	No alert	Established malnutrition	7	+++	Established malnutrition is the likely primary contributor to both muscle and cognitive impairment in the

					absence of a vascular component.
9	High alert	Alert of malnutrition	8	+++	A highly challenging clinical scenario, as aggressive intervention targeting one domain without considering the other may delay the patient's overall recovery.
10	Low alert	Established malnutrition	8	+++	Established malnutrition is the predominant driver of the combined muscle–cognitive impairment.
11	Moderate alert	Established malnutrition	9	+++	High-alert profile with simultaneous involvement of four clinical domains.
12	High alert	Established malnutrition	10	++++	The most complex presentation within this phenotype, approaching the threshold for very high alert on the overall vulnerability scale.

*: Subcategory. Alert classification: no alert: –; low alert: +; moderate alert: ++; high alert: +++; very high alert: ++++. CM. Cardiometabolic.

Supplementary Table S7. Phenotype 6 or Haematologic–cognitive alert phenotype (HC-Flag)

**Capillary haemoglobin**		Normal			2
**Sarcopenia (SARC-F)**		High Alert			0
**Cognitive screening status (RUDAS)**		suspected cognitive impairment			2
**Sub***	**Alert CM**	**Nutritional Status CC-MNA-SF**	**Score**	**Alert Clas.**	**Conceptual Interpretation**
1	No alert	Normal	4	++	anaemia and cognitive impairment likely resulting from chronic cerebral hypoxia, without other contributing factors.
2	Low alert	Normal	5	++	A mild vascular component is superimposed on anaemia-related cerebral hypoxia.
3	No alert	Alert of malnutrition	5	++	Possible shared nutritional aetiology (vitamin B12/folate deficiency), in addition to cerebral hypoxia caused by anaemia.
4	Low alert	Alert of malnutrition	6	++	Mild triple alert involving hypoxia, vascular alert, and nutritional impairment.
5	Moderate alert	Normal	6	++	A moderate vascular component is

					superimposed on anaemia-related cerebral hypoxia.
6	High Alert	Normal	7	+++	Clearly high-alert profile, with the combined effects of hypoxia and vascular disease on cognitive function.
7	Moderate alert	Alert of malnutrition	7	+++	Moderate triple-domain involvement—hypoxia, vascular alert, and nutritional impairment—requiring timely intervention.
8	No alert	Established malnutrition	7	+++	Established malnutrition as an additional nutritional factor contributing to anaemia-related cerebral hypoxia.
9	High alert	Alert of malnutrition	8	+++	Potential therapeutic dilemma due to the need for Cardiometabolic screening reduction while simultaneously increasing nutritional support.
10	Low alert	Established malnutrition	8	+++	Established malnutrition is the predominant driver of the haematologic–cognitive syndrome.

11	Moderate alert	Established malnutrition	9	+++	High-alert profile with simultaneous involvement of four clinical domains.
12	High alert	Established malnutrition	10	++++	The most complex scenario within this phenotype, associated with the greatest alert of morbidity and mortality and approaching the threshold for very high alert on the overall vulnerability scale.

*: Subcategory. Alert classification: no alert: –; low alert: +; moderate alert: ++; high alert: +++; very high alert: ++++. CM. Cardiometabolic.

Supplementary Table S8: Phenotype 7 or Haemato-Muscular Alert or HS-Flag

**Capillary haemoglobin**		Low			2
**Sarcopenia (SARC-F)**		High Alert			2
**Cognitive screening status (RUDAS)**		Normal			0
**Sub ***	**Alert CM**	**Nutritional Status CC-MNA-SF**	**Score**	**Alert Class.**	**Conceptual Interpretation**
1	No alert	Normal	4	++	anaemia and sarcopenia without any other comorbidity; Anaemia and sarcopenia may coexist and share biological and clinical determinants.
2	Low alert	Normal	5	++	Mild CM alert is added to the anaemia/sarcopenia dyad.
3	No alert	Alert of malnutrition	5	++	Possible shared nutritional origin: protein and iron deficiency.
4	Low alert	Alert of malnutrition	6	++	Mild quadruple alert: hemato-muscular, vascular, and nutritional.
5	Moderate alert	Normal	6	++	Moderate CM alert is added to the anaemia–sarcopenia dyad.
6	High Alert	Normal	7	+++	Clear high alert: greater probability of

					associated cardiometabolic morbidity and mortality.
7	Moderate alert	Alert of malnutrition	7	+++	Moderate quadruple convergence: anaemia, sarcopenia, nutritional alert, and CM alert, condition requiring care.
8	No alert	Established malnutrition	7	+++	Triple convergence: anaemia, sarcopenia, and overt malnutrition without CM alert. Pattern frequently compatible with secondary sarcopenia due to malnutrition, reinforced by probable deficiency anaemia.
9	High alert	Alert of malnutrition	8	+++	High-grade quadruple convergence alert. Possible therapeutic dilemma due to the need for restriction because of CM alert and the need for protein intake.
10	Low alert	Established malnutrition	8	+++	Quadruple convergence: anaemia,

					sarcopenia, mild CM alert, and overt malnutrition, the latter as the dominant factor of the condition.
11	Moderate alert	Established malnutrition	9	+++	High-grade quadruple convergence: anaemia, sarcopenia, moderate CM alert, and overt malnutrition, all four simultaneously affected.
12	High alert	Established malnutrition	10	++++	Quadruple convergence in its most severe degree: anaemia, sarcopenia, high CM alert, and overt malnutrition, the Most complex configuration within the conceptual matrix. of the category.

*: Subcategory. Alert classification: no alert: –; low alert: +; moderate alert: ++; high alert: +++; very high alert: ++++. CM. Cardiometabolic.

.

Supplementary Table S9: Phenotype 8 or Multidomain Alert or HSC-Flag

**Capillary haemoglobin**		Low			2
**Sarcopenia (SARC-F)**		High Alert			2
**Cognitive screening status (RUDAS)**		Suspected Cognitive Impairment			2
**Sub ***	**Alert CM**	**Nutritional Status CC-MNA-SF**	**Score**	**Alert Class.**	**Conceptual Interpretation**
1	No alert	Normal	6	++	Triple convergence: anaemia, sarcopenia, and cognitive impairment without CM or nutritional alert.
2	Low alert	Normal	7	+++	Mild CM alert is added to the triple convergence.
3	No alert	Alert of malnutrition	7	+++	Quadruple convergence: mild nutritional alert is added, with possible contribution to anaemia and cognition (B12/folate/iron).
4	Low alert	Alert of malnutrition	8	+++	Mild quintuple convergence: all five domains affected, all at a mild-to-moderate degree.
5	Moderate alert	Normal	8	+++	Quadruple convergence of factors with moderate Cardiometabolic screening.

6	High alert	Normal	9	+++	High-grade quadruple convergence: there is a greater alert of morbidity and falls.
7	Moderate alert	Alert of malnutrition	9	+++	Moderate quintuple convergence: all five domains affected.
8	No alert	Established malnutrition	9	+++	Quadruple convergence: overt malnutrition without CM alert. Compatible with a frailty syndrome of predominantly nutritional cause.
9	High alert	Alert of malnutrition	10	++++	High quintuple convergence. Possible therapeutic dilemma due to the need for restriction because of CM alert and the need for protein intake.
10	Low alert	Established malnutrition	10	++++	Overt malnutrition as the probable dominant factor of the haemato-cognitive-muscular deficits.
11	Moderate alert	Established malnutrition	11	++++	Severe quintuple convergence, approaching the threshold of very high alert on the global scale.
12	High alert	Established malnutrition	12	++++	Maximum quintuple convergence. The highest-alert scenario among the 96 possible combinations.

					Compatible with Established Potential multidimensional vulnerability requiring further assessment.

*: Subcategory. Alert classification: no alert: –; low alert: +; moderate alert: ++; high alert: +++; very high alert: ++++. CGA: Comprehensive Geriatric Assessment. CM: cardiometabolic.
